# Effects of Repeated Exposure to Ambient Cold on the Development of Inflammatory Pain in a Rat Model of Knee Arthritis

**DOI:** 10.3390/life14111428

**Published:** 2024-11-06

**Authors:** So-Hee Kim, Vishnumolakala Sindhuri, Min-Jae Koo, Seung Heon Jeon, Seungtae Kim, Sungtae Koo

**Affiliations:** 1 Korean Medicine Research Center for Healthy Aging, Pusan National University, Yangsan 50612, Republic of Korea; cableyou@naver.com; 2 Department of Korean Medical Science, School of Korean Medicine, Pusan National University, Yangsan 50612, Republic of Korea; sindhu17@pusan.ac.kr (V.S.); 67ggs@naver.com (M.-J.K.); zegehio@naver.com (S.H.J.); kimst@pusan.ac.kr (S.K.); 3 Department of Korean Medicine, School of Korean Medicine, Pusan National University, Yangsan 50612, Republic of Korea

**Keywords:** ambient cold, arthritis, weight-bearing, cold temperature, carrageenan, microglia

## Abstract

This study investigated the effects of ambient cold exposure on inflammatory pain development, synovial cytokine levels, and spinal cord glial cell activation. Male Sprague–Dawley rats (6 weeks old) were divided into Cold and RT groups. The Cold group was exposed to cold (4 ± 1 °C) for 6 h/day for 5 consecutive days, while the RT group remained at room temperature (22 ± 1 °C). On day 6, knee arthritis was induced via intra-articular carrageenan injection. Pain was assessed by weight-bearing forces (WBFs) of the affected limb. Synovial pro-inflammatory (IL-1 β, IL-6, and TNF-α) and anti-inflammatory (IL-10) cytokines were measured by ELISA, while spinal cord microglia and astrocytes activation were evaluated via immunohistochemistry. WBFs were maximally reduced 4 h post-carrageenan injection, gradually recovering afterward. Cold-exposed rats showed significantly decreased WBF on days 1 and 2 post-injection compared to the RT group. In the Cold group, synovial cytokines (IL-1β, IL-6, IL-10) were significantly elevated 4 h post-injection, with no change in TNF-α levels. Additionally, OX42-positive cells (microglia) significantly increased 1 h post-injection in the Cold group, while GFAP-positive cells (astrocyte) remained unchanged. Repeated ambient cold exposure enhances inflammatory pain development through the regulation of synovial cytokines and microglia activation in the spinal cord in carrageenan-induced knee arthritis.

## 1. Introduction

Musculoskeletal disorders are greatly influenced by weather, with exposure to cold being especially important [1,2,3]. There appears to be a direct correlation between environmental temperature and pain perception, as epidemiological studies have consistently demonstrated a higher prevalence of musculoskeletal complaints in cold climate regions [4,5,6]. In inflammatory diseases like arthritis, where patients commonly report feeling more sensitive to pain in cold weather, this relationship is particularly noticeable [7,8]. Several studies have shown that people who work repeatedly in cold environments have a higher risk of shoulder [3,9,10], knee [3], lower back, and neck pain [11,12,13] than at normal temperatures.

Inflammatory pain and cold exposure are related in a way that involves intricate physiological reactions. In an effort to maintain thermal homeostasis, the body experiences increased muscle tension in cold temperatures, as shown by elevated electromyography (EMG), which measures electrical activity in muscle activity [14]. Local inflammatory responses and pain sensitivity can be altered by this increased muscle activity in conjunction with cold-induced vasoconstriction [15]. Additionally, certain thermosensitive ion channels, especially TRPM8 [16] and TRPA1 [17], are activated by cold exposure and have the ability to modify nociceptive signaling and inflammatory processes.

The effects of cold exposure on arthritis go beyond peripheral mechanisms and involve reactions in the central nervous system. The immune cells called microglia, which are found in the brain and spinal cord, are essential for processing pain and causing neuroinflammation [18]. These cells release pro-inflammatory mediators [19] in response to a variety of environmental stressors, such as temperature changes, which can intensify pain sensitivity. Microglial activation is a crucial mechanism in pain sensitization, according to recent research, especially in situations where environmental stressors may alter inflammatory responses [20].

The possible effects of cold exposure prior to the onset of arthritis are still poorly understood, despite the fact that prior studies have looked at the effects of weather change (decreased by 7 °C from 22 °C) on chronic pain [21] and the effects of cold treatment (4 °C ice bath) on the acute model of arthritis [22]. Given that weather factors that precede the onset of disease may affect the susceptibility and progression of that disease, this represents a critical knowledge gap. For those who are at risk of developing arthritis and reside in cold climates, knowing these pre-disease environmental factors may offer important insights for preventive measures.

Given the correlation between cold exposure and the onset of inflammatory pain, we hypothesized that repeated exposure to cold temperatures (4 °C) before arthritis induction would increase inflammatory responses and worsen pain sensitivity. In a rat model of carrageenan-induced knee arthritis, we investigated the effects of repeated exposure to ambient cold on the development of inflammatory pain, paying particular attention to both peripheral (synovial cytokine levels) and central (spinal cord glial cell activation) mechanisms.

It may be very helpful to develop prevention and treatment strategies for arthritis and other inflammatory diseases if we can comprehend how prior cold exposure influences inflammatory pain [23]. This knowledge could also help to clarify why certain individuals are more prone to experiencing severe arthritic symptoms during colder months [24].

## 2. Materials and Methods

### 2.1. Animals

Adult male Sprague–Dawley rats (SD), weighing 180–200 g, were purchased from Hyochang Science (Daegu, Republic of Korea). The rats were housed 2 per cage with soft bedding, cared for, and maintained under a 12-h light/dark cycle at a constant temperature of 22 ± 1 °C and with relative humidity (50 ± 5%). The rats were allowed free access to food and water and were used for animal behavior test (*n* = 16, 8 per group), evaluation of synovial fluid cytokines (*n* = 36, 6 per group for each time point), and immunohistochemical analysis (*n* = 24, 3 per group for each time points). The rats were given a week to get used to the lab setting. The same researcher carried out the procedures at regular times each day and kept noise levels below 60 dB in order to reduce stressors during the experimental period. Access to the laboratory was also limited in order to reduce outside stimuli. This study was approved by the Ethical Committee for Animal Study at Pusan National University (PNU-2019-2238). We used SigmaPlot 14.0 software to calculate the sample size for the behavioral test. We set a minimum detectable difference in means of 10, an expected standard deviation of residuals of 6.5, a number of groups of 2, a significance level (α) of 0.05, and a power (1-β) of 0.8, which meant that there should be 8 animals per group.

### 2.2. Exposure to Ambient Cold and Induction of Inflammatory Pain

**Figure 1:**
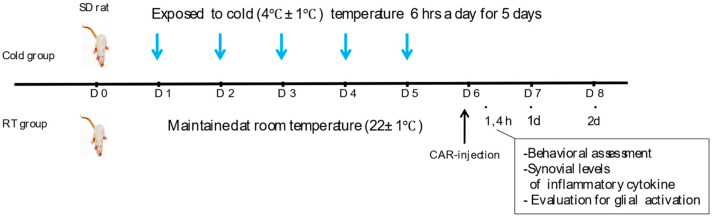
Schematic diagram summarizing the experimental timeline of the study. Male Sprague–Dawley rats, weighing 180–200 g are randomly assigned to one of the Cold and RT groups (*n* = 8 per group). Cold group is exposed to cold temperature (4 ± 1 °C) 6 h a day for 5 consecutive days, and RT group is housed at room temperature (22 ± 1 °C) without change in temperature. After carrageenan injection, weight bearing forces (WBFs) of the affected hind limb, the levels of inflammatory cytokines in synovial fluids, and glial activation in the spinal cord were investigated.

After a period of adaptation, rats were randomly assigned to an experimental (Cold group) or a control group (RT group). The Cold and RT groups were kept in identical conditions, with a 12-h light/dark cycle and relative humidity of 50 ± 5 percent. Ad libitum food and water were given, and the only distinction between the groups was the temperature (4 °C vs. 22 °C). For the cold exposure, the animals were housed in a noise-free cold chamber. The Cold group (*n* = 8) was exposed to the noxious cold temperature (4 ± 1 °C) 6 h a day for 5 days, respectively, with relative humidity (50 ± 5%); an RT group was kept consecutively without any thermal changes during the same period (*n* = 8). And then, on the sixth day, all rats were gas-anesthetized with 3% isoflurane (JW Pharmaceutical Corp, Seoul, Republic of Korea), and followed to inject into the right knee joint with 0.1 mL of 2% Carrageenan (Sigma, St. Louis, MO, USA) for the induction of acute knee arthritis (Figure 1). The cold exposure temperature (4 ± 1 °C) was selected based on previous studies investigating cold-induced inflammatory responses [25,26]. This temperature has been shown to effectively induce cold stress responses while maintaining ethical considerations for animal welfare.

### 2.3. Behavioural Test

The level of inflammatory pain was assessed by measuring the weight-bearing forces (WBFs) of the affected limb of a walking rat at 4 h, day 1, and 2 post-induction of arthritis [26]. Briefly, each rat was placed into the starting line of an acrylic-, rectangular apparatus (length × height × width: 80 × 10 × 10 cm) with a pressure sensor plate at the middle of the path. While a rat was walking through the path, the weight load of the affected leg of the rat was measured on the plate and monitored using a CED Power 1401 A/D board (CED 1401 Cambridge Electronic Design, Cambridge, UK) and Spike2 data acquisition software (6.18) (Cambridge Electronic Design, Cambridge, UK). The apparatus used for WBF measurements has been validated in previous studies [27,28,29,30,31]. For measurement accuracy, three readings were taken per animal, and the mean value was used for analysis. The WBF was converted to a weight-bearing ratio according to the following formula: Weight-Bearing Ratio (%) = (Post-injection value/pre-injection value) × 100.

The measurement was performed before (for baseline) and after the carrageenan injection. An independent researcher who was blind to the group assignments conducted the behavioral testing. Weight-bearing force (WBF) measurements were conducted with the data collector blind to group assignment.

### 2.4. Cytokine Analysis of Synovial Fluid

To evaluate the cytokine levels of interleukin IL-1β, IL-6, IL-10, and tumor necrosis factor-α (TNF-α) in synovial fluid, the rats were temporarily anesthetized by inhalation of 2% isoflurane (JW Pharmaceutical Corp., Seoul, Republic of Korea) at 4 h, 1 day, and 2 days after induction of arthritis. Synovial fluid collection was performed under sterile conditions to minimize contamination. Synovial fluid was collected by first injecting 150 µL of saline intra-articularly using a 30-gauge screw needle (Feeltech, Exeter, UK; 1 mL), and then aspirating 100 µL of the synovial fluid and saline back. Synovial fluids were centrifuged at 10,000× *g* for 30 min at 4 °C, and the supernatants were collected for multiplex cytokine array analysis (R&D System Inc., Minneapolis, MN, USA) that quantitatively designed for IL-1β, IL-6, IL-10, and TNF-α.

### 2.5. Immunohistochemistry

Spinal cord sections were extracted quickly from naive (non-arthritic rats), Cold, and RT groups, and washed in 0.05 M phosphate-buffered saline (PBS), immediately fixed in 4% paraformaldehyde for 6–8 h, and then immersed in 30% sucrose at 4 °C for 48 h. They were then sectioned at 30–50 µm thickness on a freezing microtome (CM3050S; Leica Biosystems, Wetzlar, Germany). The sections were blocked with CAS-block (Invitrogen-Molecular Probes, Inc., Camorillo, CA, USA) for 10 min at 25 °C and incubated with mouse anti-OX-42 (1:1000; Abcam Inc., Cambridge, UK), mouse anti-glial fibrillary acidic protein (GFAP) (1:400; Millipore, Temecula, CA, USA). After three washes with PBS, they were incubated with the biotinylated goat anti-mouse secondary antibody (1:100; Vector Laboratories Inc., Burlingame, CA, USA) for 2 h at room temperature. To guarantee antibody specificity, we included isotype-matched control antibodies, secondary antibody-only controls, and negative controls that excluded primary antibodies in our immunohistochemical analysis. To ensure reproducibility, three separate repetitions of each experiment were carried out under the same setup.

### 2.6. Statistical Analyses

Statistical analysis was performed with GraphPad Prism 10 for Windows (10.3.0) (GraphPad Software, Boston, MA, USA) Statistical significance was determined by two-way analysis of variance (ANOVA) with Tukey’s post hoc test. All data are expressed as the mean ± standard error of the mean and a probability of *p* < 0.05 was accepted as statistically significant. Graphs were generated using Prism 10 for Windows (10.3.0) (GraphPad Software, Boston, MA, USA).

## 3. Results

### 3.1. Effect of Cold Exposure on the Development of Inflammatory Pain

**Figure 2:**
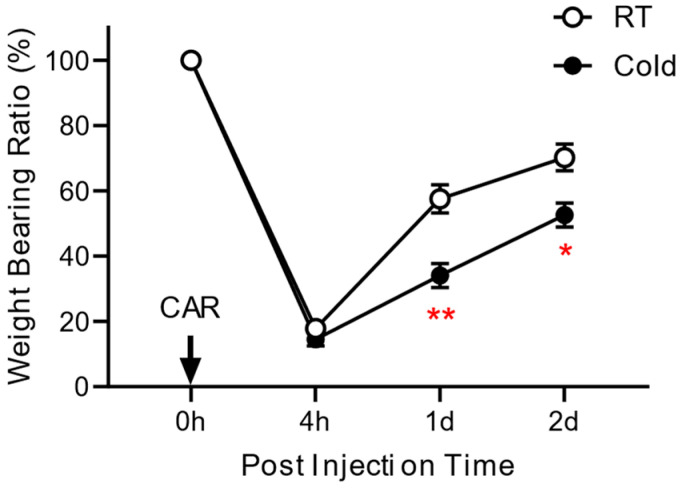
Effect of repeated exposure to ambient cold on the pain behavior. The weight-bearing ratio (WBR) of the affected hind limb in the Cold and RT groups was examined at 4 h, 1 d, and 2 d after carrageenan (CAR) injection. The WBR was calculated as the percentage of WBF of the post-injection value over the pre-injection value, and data are expressed as mean ± the S.E.M. * *p* < 0.05, ** *p* < 0.01, compared to the RT group.

To determine whether ambient temperature influences the development of inflammatory pain, we divided rats into two groups: the Cold-exposed group (Cold group, *n* = 8) and a group without thermal change (RT group, *n* = 8). Figure 1 presents the schematic diagram, showing the experimental procedure of this study. As shown in Figure 2, the weight-bearing forces of all groups showed maximal impairment at 4 h and then gradually recovered. At 4 h after the injection of carrageenan, the weight load of the affected leg was 15.2 ± 1% for the Cold group and 20.6 ± 2% for the RT group. Repetitive exposure to the cold resulted in the delay of pain alleviation of 36.3 ± 1% and 17.3 ± 5% on day 1 and 31.4 ± 2% and 15.6 ± 2% on day 2, compared to those of RT groups. There are statistically significant differences between the Cold and RT groups on day 1 (*p* = 0.0039) and day 2 (*p* = 0.025). Repeated stimuli of different temperatures showed different responses to pain-related behavior, suggesting a positive link between exposure to ambient cold and the development of inflammatory pain.

### 3.2. Synovial Levels of Inflammatory Cytokines Altered by Cold Exposure in Carrageenan-Induced Knee Arthritis

**Figure 3:**
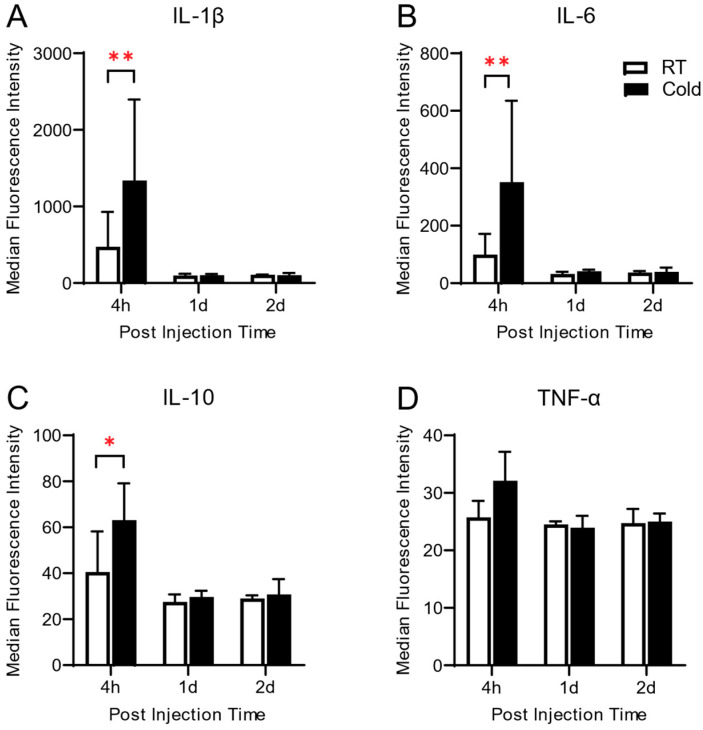
Effect of repeated exposure to ambient cold on synovial levels of inflammatory cytokines. The levels of pro-inflammatory (interleukin (IL)-1β (**A**), IL-6 (**B**) and tumor necrosis factor-α (TNF-α) (**D**)) and anti-inflammatory (IL-10 (**C**)) cytokines were measured in synovial fluid samples by using ELISA in the Cold and RT groups (*n* = 6 each) at 4 h, 1 d and 2 d after carrageenan injection. Data are expressed as mean fluorescence intensity with S.E.M. (* *p* < 0.05, ** *p* < 0.01 vs. RT group).

Next, to investigate whether cold exposure before acute inflammation induces any change in the local inflammatory response, we measured the synovial levels of pro-inflammatory (Interleukin (IL)-1β, IL-6 and tumor necrosis factor (TNF)-α, and anti-inflammatory (IL-10) cytokine. Synovial levels of IL-1β, IL-6, and IL-10 were significantly increased in the Cold group at 4 h post-arthritic induction (*p* < 0.001, *p* < 0.001, and *p* = 0.0038, respectively), compared to those of the RT group, and fell to the basal level of RT group after 1-day post-induction (Figure 3). There was a slight increase in TNF-α at 4 h but no statistical difference was observed.

### 3.3. Effect of Cold Exposure on Spinal Glial Activation in Carrageenan-Induced Knee Arthritis

**Figure 4:**
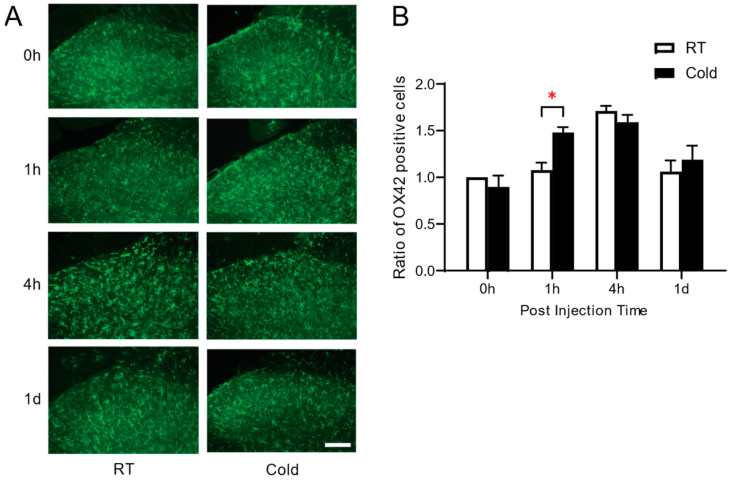
Effect of repeated exposure to ambient cold on the expression of OX-42 positive cells in spinal cord. (**A**) Representative images of OX-42 positive cells in spinal cord, (**B**) the ratio of number of OX-42 positive cells in Cold group, compared to RT group were analyzed at the time courses of 0 h, 1 h, 4 h and 1 d. Scale bar = 40 µm. * *p* < 0.05, vs. RT (Two-way ANOVA followed by Tukey’s method). Data are presented as mean ± S.E.M.

**Figure 5:**
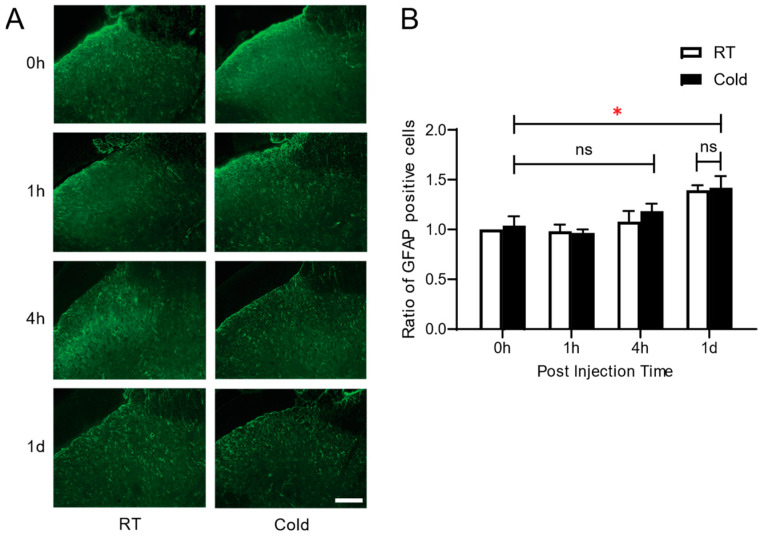
Effect of repeated exposure to ambient cold on the expression of GFAP-positive cells in spinal cord. (**A**) Representative images of GFAP positive cells in spinal cord, (**B**) the ratio of number of GFAP positive cells in Cold group, compared to RT group were analyzed at time courses of 0 h, 1 h, 4 h, and 1 d after carrageenan injection. Scale bar = 40 µm. * *p* < 0.05, 0 h vs. 1 d (Two-way ANOVA followed by Tukey’s method). Data are presented as mean ± S.E.M.

To examine the effect of cold exposure prior to the development of inflammatory pain on glial cells, we performed immunohistochemistry to compare the activation of microglia (OX-42) and astrocytes (GFAP) in the dorsal horn of the spinal cord in the Cold and RT groups at 1 h, 4 h, and 1 d. The immunostaining study revealed that both groups exhibited an increase in OX-42 positive cells in the early phase (at 1 h, and 4 h) compared to naïve non-arthritic rats (0 h), and fell to those of basal level at 1 d (Figure 4A,B). Significant differences were observed at 1 h post arthritic induction (*p* = 0.0042 * RT vs. Cold). In contrast with the level of microglial activation, there was no signification change in astrocytes in both RT and Cold groups (Figure 5A,B). There were no significant differences in the number of GFAP-positive cells between the Cold and RT groups across all four measurement times, indicating that cold exposure did not result in different levels of astrocyte activation between the groups at any specific time point. However, within each group, there was a significant increase in the number of GFAP-positive cells at 1 day (1 d) compared to the baseline (0 h). This finding suggests that irrespective of cold exposure, both groups experienced a significant increase in astrocyte activation over time following the inflammatory stimulus.

## 4. Discussion

The relationship between weather factors and arthritic symptoms is an interesting issue, but studies on the causality remain inconclusive up to now. Some studies have demonstrated that ambient cold aggravates arthritic symptoms such as pain sensitivity and inflammatory responses in patients [32,33] and experimental animals [17,34], whereas others fail to find the causal relationship between them. We recognize that there are differences in the research on the relationship between cold exposure and arthritic symptoms. These discrepancies most likely result from a number of methodological differences between studies. First, there are significant differences in the intensity of cold exposure; some studies used acute exposure (4 °C ice bath for 20 min [22], immersed in 10 °C water for 5 min [17], exposed to 10 °C for 1 h [34]) while others looked at chronic effects (weeks to months [7,33]). Second, there was a considerable temperature variation (4–10 °C), which could have set off different physiological reactions. Third, a range of animal models (rats versus mice, different strains) that may respond differently to cold stress based on the species. Furthermore, the inflammatory cascade may have been affected differently by the various studies’ use of varying cold exposure timings in connection to the introduction of inflammatory stimuli. Understanding these methodological differences helps to explain the findings in the literature that do not seem to fit together. In this study, we currently focused on the relationship between cold exposure and the development of inflammatory pain.

To investigate thoroughly the effect of cold exposure on pain-related behavior, two factors (relative humidity, and air pressure), which are known to be responsible for arthritic-pain intensity [35,36] were maintained in the same condition during experiments. We also measured serum cortisol levels of the rats before induction of acute arthritis, because unusual changes in cold- (4 ± 1 °C) temperature may cause unpleasant sensations in experimental animals. We confirmed that there is no change in serum levels of cortisol by temperature change.

Sugama et al. [37] have observed that cold stress induced significant increases in plasma ACTH and corticosterone (CORT) levels and morphological microglial activation during two h of cold stress. In the study, the levels of ACTH and CORT were measured from plasma samples from rats, which were sacrificed immediately after cold stress of 4 °C. However, in our study, the effects of 6 h-cold exposures were measured after the body temperature of rats was maintained back to the normal temperature. So, the mechanisms of body homeostasis might induce the regulation of the hormone level to daily conditions. Cortisol is a more abundant glucocorticoid in humans, whereas corticosterone is much more in rodents. Unfortunately, we did not test the corticosterone during the experiments, but the difference in hormone levels is predicted to be insignificant due to the homeostatic mechanism.

Results from the weight-bearing test showed a greater decrease in painful behavior and delayed recovery in the cold-exposed group than those of the RT group (Figure 2), indicating cold exposure, itself might be a factor to influence on occurrence and pain severity of arthritis to some degree.

The inflammatory response in the peripheral and nervous systems is known to play a key role not only in the initiation but also in the persistence of pathologic pain states [38]. In this study, there was a significant increase in the synovial levels of pro- and anti-inflammatory cytokine in cold-exposed rats at 4 h, compared to that of the RT group, and then declined very rapidly (Figure 3). No significant difference was observed in the level of TNF-α between the Cold and RT groups. This finding suggests that cold exposure on inflammatory response did not affect any systemic inflammatory effects in carrageenan-induced knee joint inflammation. As our knowledge, TNF-α is considered a good mediator of correlating with pathogenesis in rheumatoid arthritis [32], in carrageenan-induced acute inflammation [39,40], and in the chronic arthritic condition [41,42,43] in the early phase of inflammation.

The lack of notable changes in TNF-α levels 4 h after exposure calls for careful analysis. TNF-α is usually an early response cytokine, reaching its peak levels one to two hours after an inflammatory stimulus. It is possible that our 4-h time point missed the first spike in TNF-α. Furthermore, rather than TNF-α-dependent inflammation, cold exposure may preferentially activate the IL-1β and IL-6 pathways. Another possibility is that TNF-α levels were already normalized by four hours due to compensatory anti-inflammatory mechanisms. Additionally, we may need to look into how the TNF-α level reacts to cold exposure earlier than 4 h. To completely comprehend the distinct temporal dynamics of different inflammatory mediators in cold-induced inflammation, more investigation is required.

The significant increase in OX42-positive microglia in the cold-exposed group shortly after carrageenan injection (Figure 4) suggests that repeated exposure to noxious cold sensitizes the central nervous system (CNS) to inflammatory stimuli, leading to enhanced neuroinflammatory responses. Microglia, as the primary immune cells in the CNS, are known to rapidly respond to environmental stressors, such as cold, by becoming activated and releasing pro-inflammatory mediators that contribute to pain hypersensitivity. In contrast, the lack of significant change in GFAP-positive astrocyte expression (Figure 5) indicates that astrocytes may not play a prominent role in the early phase of inflammation or acute inflammatory pain induced by cold exposure. Our cold exposure paradigm is acute, which is probably why there is not much astrocyte activation during the period of increased pain behavior. In general, astrocytes react to inflammatory stimuli more slowly than microglia; activation usually becomes apparent 24 to 48 h later. Additionally, persistent or intense inflammatory stimuli are frequently necessary for astrocyte activation. We might not have experienced enough cold exposure to cause robust astrogliosis. In cold-induced inflammatory pain, this temporal variation in glial cell activation points to a sequential response in which microglial activation comes before astrocytic involvement. This finding suggests that while microglia are more sensitive to acute inflammatory triggers, astrocytes may require prolonged or more severe inflammatory conditions to become activated.

We propose several ways in which cold exposure may increase the sensitivity of the central nervous system to inflammatory pain. Exposure to cold most likely activates thermosensitive ion channels (e.g., TRPM8, TRPA1) in peripheral neurons, increasing the amount of nociceptive input that reaches the spinal cord. This increased input may trigger microglial activation through the release of neurotransmitters such as substance P and CGRP, along with damage-associated molecular patterns (DAMPs). These factors trigger microglial activation through specific pattern recognition receptors (e.g., TLR4, P2X7R), initiating a signaling cascade involving NF-κB pathway activation. Activated microglia then release pro-inflammatory mediators like IL-1β and IL-6 through the NLRP3 inflammasome pathway, which can sensitize nearby neurons and enhance pain signaling. Additionally, cold stress may cause the sympathetic nervous system to release norepinephrine, which can change immune cell function and inflammatory responses. Furthermore, cold exposure results in further influences on inflammatory reactions, leading to a boost in immune responses triggered by modified proteins like IgG [44] and the emergence of reactive oxygen species (ROS) [45] in arthritis.

It is possible to consider our findings regarding the effects of environmental factors, particularly exposure to cold, on arthritis pain in the larger framework of chronic pain conditions like fibromyalgia. Similar to arthritis, fibromyalgia symptoms can be influenced by environmental factors, such as temperature fluctuations [46]. The biopsychosocial model of pain in fibromyalgia, in which several factors contribute to symptom severity [47], is similar to the intricate relationship between environmental stressors and pain mechanisms that we observed in our study. Gaining an understanding of these parallel mechanisms may help identify common pathways of environmental influence on chronic pain conditions.

Our research has a number of significant ramifications for comprehending and managing inflammatory pain brought on by cold. First, they propose that arthritis patients may benefit most from preventative anti-inflammatory therapies prior to exposure to cold temperatures. Second, the discovery of early microglial activation raises the possibility that glial cell targeting could be a successful therapeutic approach. Third, our findings suggest that controlling inflammatory pain may require stable ambient temperatures. Patients with cold-sensitive arthritis may benefit from tailored interventions developed using these insights.

Our study has a number of limitations, which we acknowledge. First, although we measured cortisol, we did not measure corticosterone, which would have been more suitable for evaluating the stress response in rodents. Second, we cannot totally rule out stress’s impact on our outcomes, even in the face of efforts to reduce it. Third, our brief (6-h) exposure period might not accurately represent the effects of long-term cold exposure that arthritis sufferers usually encounter. Fourth, the long-term effects of cold exposure need further research, as our study concentrated on acute inflammatory responses. Finally, we acknowledge that the results we obtained from young, healthy male rats might not apply entirely to older or female subjects or to people who already have health issues.

Future research should address a number of significant questions that our findings raise: (1) analyzing earlier time points (0–4 h) to capture the full temporal profile of inflammatory mediators, especially TNF-α; (2) examining the effects of chronic cold exposure on glial activation and inflammatory pain; (3) investigating sex differences in cold-induced pain sensitization; (4) assessing potential therapeutic interventions that target specific mechanisms identified in this study; and (5) extrapolating these findings to human subjects with pre-existing inflammatory conditions. These research directions could significantly advance our understanding of environmental influences on inflammatory pain and lead to more effective therapeutic strategies.

The present study is one of a few to describe the relationship between cold exposure and the development of inflammatory pain, and the first report to suggest experimental evidence. In conclusion, our research shows that exposure to cold specifically promotes the development of inflammatory pain via a number of mechanisms, including (1) early spinal microglia activation, (2) quick elevation of pro-inflammatory cytokines IL-1β and IL-6, and (3) heightened sensitivity to peripheral inflammatory stimuli. A particular temporal sequence of cellular responses in cold-induced pain sensitization is suggested by these alterations, which take place within hours of cold exposure and come before notable astrocytic activation.

## Data Availability

The raw data supporting the conclusions of this article will be made available by the authors on request.
